# MUSCLE MITOCHONDRIAL FUNCTION PREDICTS COGNITIVE IMPAIRMENT AND IS ASSOCIATED WITH PET AND BLOOD BIOMARKERS OF AD

**DOI:** 10.1093/geroni/igad104.0585

**Published:** 2023-12-21

**Authors:** Qu Tian, Murat Bilgel, Keenan Walker, Abhay R Moghekar, Susan Resnick, Luigi Ferrucci

**Affiliations:** National Institute on Aging, National Institutes of Health, Baltimore, Maryland, United States; National Institute on Aging, National Institutes of Health, Baltimore, Maryland, United States; National Institute on Aging, National Institutes of Health, Baltimore, Maryland, United States; Johns Hopkins University, Baltimore, Maryland, United States; National Institute on Aging, National Institutes of Health, Baltimore, Maryland, United States; National Institute on Aging, National Institutes of Health, Baltimore, Maryland, United States

## Abstract

Mitochondrial dysfunction is a hallmark of aging. Whether mitochondrial dysfunction is related to future cognitive impairment and biomarkers of Alzheimer’s disease (AD) is unknown. In the Baltimore Longitudinal Study of Aging, we used Cox regression to examine the association of skeletal muscle mitochondrial function (post-exercise recovery rate of phosphocreatine, kPCr) via MR spectroscopy with future MCI or dementia over an average follow-up of 3.3 years among 460 initially cognitively normal participants (mean age=76.8±11.0 years, 56.7% women, 22.4% Black). We also examined associations with PiB-distribution volume ratio (DVR) for β-amyloid (Aβ)(n=115), Flortaucipir (FTP)-standardized uptake value ratio (SUVR) for tau(n=70), and blood biomarkers of AD pathology (Aβ42/Aβ40 ratio,n=299; pTau181,n=505), neuronal injury (NfL)(n=299), and astrocyte-mediated neuroinflammation (GFAP)(n=299) using linear regression. After adjustment for age, sex, and race, each SD higher kPCr (i.e. 0.005) was associated with 45% lower hazards of developing cognitive impairment (19 MCI, 4 dementia) (p=0.04). In the PET sample, each SD higher kPCr was associated with 51% lower odds of being PiB positive(p=0.003). Higher kPCr was specifically associated with lower DVR in precentral and postcentral gyri, superior parietal lobe, and pallidum (FDR-adjusted p<0.05). In samples with blood biomarkers, higher kPCr was associated with lower GFAP(p=0.01) and showed a trend toward significance with lower pTau181(p=0.08). Preserved skeletal muscle mitochondrial function is associated with reduced risk of cognitive impairment and PET and blood biomarkers of AD. Future studies are needed to investigate the role of mitochondrial function of the central nervous system in dementia risk and the progression of AD pathologic change.

